# Multicystic Hepatocarcinoma Mimicking Liver Abscess

**DOI:** 10.1155/2013/374905

**Published:** 2013-01-13

**Authors:** Evangelos Falidas, Angelos Pazidis, Georgios Anyfantakis, Konstantinos Vlachos, Christina Goudeli, Constantinos Villias

**Affiliations:** ^1^1st Department of Surgery, 417 NIMTS Veterans Hospital of Athens, 10-12 Monis Petraki, 11521 Athens, Greece; ^2^Department of Surgery, Florina General Hospital, Egnatias 9, 53100 Florina, Greece; ^3^Department of Radiology, 417 NIMTS Veterans Hospital of Athens, 10-12 Monis Petraki, 11521 Athens, Greece

## Abstract

The diagnosis of hepatocellular carcinoma (HCC) became easier in relation to the improved radiological examinations; however, the neoplasm may occur under atypical presentations mimicking other benign or malignant processes. Multicystic HCC mimicking a liver abscess associated with septic-type fever and leukocytosis is rare, has a poor prognosis, and poses diagnostic and therapeutic dilemmas. We present the case of an 80-year-old patient, who presented with fever, leukocytosis, and large cystic masses involving right and left lobes of the liver initially considered abscesses and finally diagnosed as HCC after open drainage and liver biopsy. Although the patient died on the tenth postoperative day due to pulmonary oedema, the authors emphasize the high index of suspicion needed in the diagnosis of this unusual presentation of HCC.

## 1. Introduction

Hepatocellular carcinoma (HCC) is the fifth more common neoplastic process and the third leading cause of cancer-related deaths worldwide [1]. Although the diagnosis of HCC is easier to be established mainly in relation to the improved radiodiagnostic examinations, the neoplasm may occur under atypical presentations mimicking other benign or malignant processes [2]. Multicystic HCC mimicking a liver abscess associated with septic-type fever and leucocytosis is rare, has a poor prognosis, and poses diagnostic and therapeutic dilemmas [3]. We present the case of an 80-year-old patient presented with fever, leucocytosis, and large cystic masses involving right and left lobes of the liver initially considered abscesses and finally diagnosed as HCC after open drainage and liver biopsy.

## 2. Case Presentation

**Figure 1:**
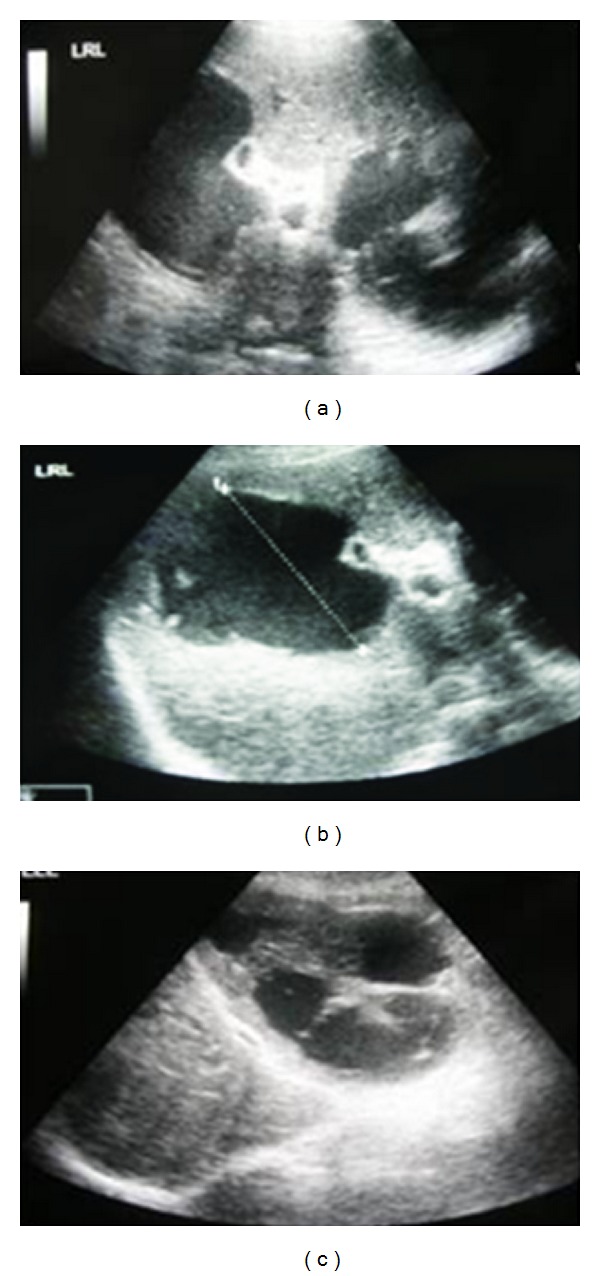
Abdominal ultrasound demonstrating cystic lesions in the right lobe of the liver that also involves almost the entire left liver lobe.

**Figure 2:**
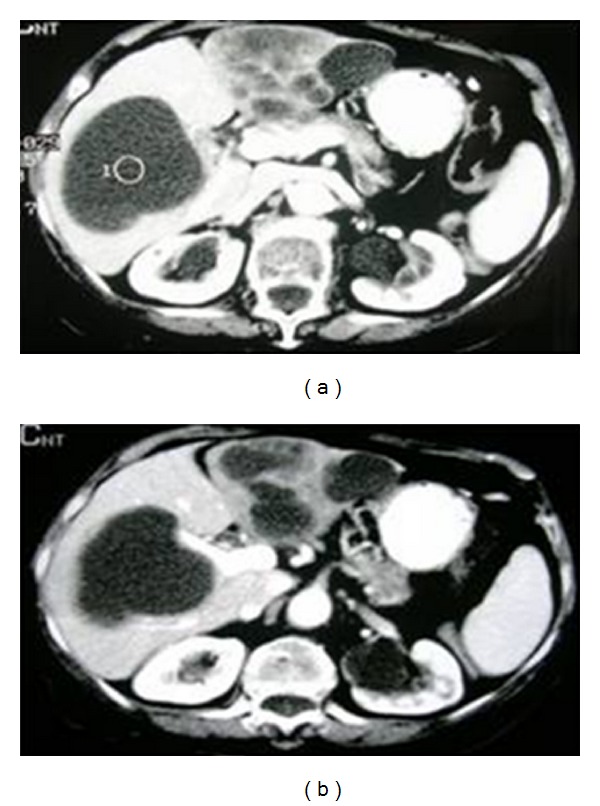
CT scan demonstrating cystic lesions or hypodense hepatic masses covering almost the entire part of the hepatic parenchyma.

An 80-year-old woman came to the Emergency Department reporting 4-day lasting fever (>39°C), successfully treated with paracetamol, and abdominal pain mainly located in the right hypochondrium and the epigastrium. Her medical history included cholecystectomy followed by endoscopic retrograde cholangiopancreatography (ERCP) six months ago, large bowel diverticulitis diagnosed 2 years prior to the observation, and atrial fibrillation under treatment with acenocoumarol and digitoxin. She mentioned no alcohol consumption, while she was also negative for HBV and HCV infection. Upon examination, the pain was mainly located at the right hypochondrium with rebound tenderness, while a painful liver mass was also palpated. Temperature was also high (38,4°C). Abnormal laboratory findings included leukocytosis (19,000 K/*μ*L), anaemia (Hb: 9,1 g/dL), hyperuricemia (urea: 102 mg/dL [normal: 7–21 mg/dL], creatinine: 2,4 mg/d [normal: 0,5–1,4 mg/dL]), C-reactive protein (CRP: 12,4 mg/dL), and significant alterations of the hepatic biology (gamma-GT: 230 IU/L [normal: 8–78 IU/L], ALP: 301 IU/L [normal: 38–126 IU/L], ALT: 75 IU/L [normal: 7–56 IU/L], total bilirubin: 3,4 mg/dL [normal: 0,2–1,3 mg/dL], and direct bilirubin: 2,6 mg/dL [normal: <0,3 mg/dL]). Coagulation parameters were also abnormal (INR: 4,8). X-ray series of the chest and of the abdomen were normal. Abdominal ultrasound (US) revealed a large thin-walled cystic mass on the right liver lobe and multiple cystic masses involving almost the entire left hepatic lobe. Some of the cysts presented an irregular outline (Figures 1(a), 1(b), and 1(c)). Little amount of free fluid into the Douglas space and splenomegaly was also found. Abdominal computed tomography scan (CT) described multiple hypodense loci on both hepatic lobes (Figures 2(a) and 2(b)). No signs of diverticulitis were observed. Taking into consideration ultrasound and CT findings, diagnosis of liver abscess was initially posted. Fluid resuscitation was performed, and empirical antibiotic treatment (ciprofloxacin and metronidazole), vitamin K, and two units of plasma were administered. Acenocumarol was replaced with enoxaparin sodium. Blood and urine cultures were also taken on admission.

Clinical condition was improved during the first 72 hours after admission, although leucocytosis (±1,500 K/*μ*L/day), fever and CRP (9,8 mg/dL) remained steadily elevated. The hepatic biochemistry was gradually worsening (ALP up to 519 IU/L, *γ*-GT up to 946 IU/L). Blood and urine cultures resulted in sterility. Evaluation for parasitic (antiechinococcus, antiamoebic antibodies) infection was also negative. Among tumor markers, only Ca 19-9 and Ca-125 were slightly elevated (139 U/mL [normal: <40 U/mL] and 201 U/mL [normal: <35 U/mL], resp.), while  *α*FP was within normal range (normal: <35 U/mL). Repetitive abdominal ultrasounds did not reveal particular changes of the aspect of the hepatic lesions.

Percutaneous drainage was initially considered, although the number of the lesions made us choose an open drainage with liver biopsies. Coagulation parameters were normalized (INR: 1,4). Upon laparotomy, multiple and large cystic lesions of the liver surface were identified in a context of a diffuse shrinkage of the parenchyma. The cystic content (yellowish, serum liquid) was initially aspirated and the cystic wall was removed. Multiple biopsies were taken from the cystic borders including liver parenchyma as well as from the remaining liver parenchyma. Cytology examination revealed only neutrophils.

During immediate postoperative course, fever decreased while leukocytosis remained steadily elevated and gradually increasing (up to 27,800 K/*μ*L). Temperature remained normal during the rest of her hospitalization. Although postoperative course improved, she died on the tenth postoperative day because of pulmonary oedema. Histological examination described complete destruction of the hepatic architecture and presence of cells with large vacuole-like, small, or multiple nuclei and dense cell membranes. Mitoses were multiple and atypical. Stains for PAS-D and Alcian Blu were negative. Immunohistochemical stain was positive for EMA, Vimentin, AFP, and Keratin 8 and negative for CEA, Keratin 5/6, and HMB45. Diagnosis of hepatocellular carcinoma was posted.

## 3. Discussion

Hepatocellular carcinoma (HCC) is the most common primary hepatic neoplasm (80–90%). About 10% of patients with liver cirrhosis may develop HCC while 90% of primary HCC seem to be related with a chronic hepatopathy [4, 5]. HCC is strongly connected with viral B or C hepatitis, chronic hepatitis, nitrosamine or aphlatoxin consumption, alcoholic cirrhosis, and hemochromatosis. HCC may be asymptomatic or may appear with hepatomegaly with or without splenomegaly, ascites, superficial venous dilatation, or jaundice. Abdominal mass, abdominal pain or discomfort, anorexia, nausea, vomiting, and weight loss are commonly reported [6]. Association of fever and leukocytosis with HCC is uncommon but not rare and sometimes associated with tumor infection [7]. In many cases the tumor is incidentally discovered upon the investigation for anemia, weight loss or for treating general cirrhotic symptoms [8]. Hepatocellular carcinoma may coexist with cholangiocarcinoma or may present sarcomatous degeneration [9]. Hepatic cystic masses associated with intermittent pyrexia and leukocytosis strongly raise the suspicion of a hepatic abscess or pseudotumour. Hepatic abscesses may be attributed to biliary inflammation (lithiasis) or portal bacteraemia as a result of bowel inflammation [10]. Early detection of HCC has been increased in patients with chronic viral and nonviral hepatitis due to screening programs with the periodical application of alpha-fetoprotein test and US every six months [11, 12]. US examination usually reveals a hypoechogenic, homogeneous, and well-defined lesion for small tumors (<3 cm) or a hyperechogenic and heterogeneous lesion for greater tumors (>3 cm) due to central necrosis, fat, and sinusoidal dilatation [13]. At CT, HCC usually appears hypervascular (80%) with maximum enhancement during the arterial phase. HCC may present as hyperdense, heterogeneously hyperdense, or isodense mass [2, 14, 15]. This hypervascular aspect has high predictive value for HHC in cirrhotic patients. Peripheral enhancement may be seen in delayed phases due to fibrotic capsule. Mosaic diffuse pattern may be observed in the portal phase when phenomena such as necrosis, fibrosis, hemorrhages, and arteriovenous anastomosis occur [16, 17].

Giant, infiltrating, bleeding, calcified, exophytic, and hypovascular HCC and HCC with spontaneous regression or with fat are unusual radiological presentations of HCC and constitute less than 20% of the cases [13].

Cystic HCC in the context of a noncirrhotic liver is extremely rare [18–20]. In triphasic contrast CT it is presented as a hypodense mass—either unilocular or, more commonly, multilocular—with contrast enhancement of the solid parts in the arterial phase and typical washing out of the contrast in the portal phase [2, 15].

Magnetic resonance imaging (MRI) seems to be complementary to CT in diagnosing HCC offering a more complete cholangiographic description, although not always available, as in our case [21].

On the other hand, liver abscess may be seen as a hypoechogenic mass with septa and diaphragms. CT scan often describes hypodense—with peripheral ring contrast enhancement—or mosaic appearance of liver abscesses separated by septations [22]. This imaging presentation may cause further difficulties in the diagnosis of cystic HCC, as small HCCs may present enhancement on the portal vein phase due to delayed perfusion of pseudocapsule, thus additionally mimicking unilocular abscess [2]. High fever and leukocytosis have been well described as symptoms of HCC [7]. These clinical findings and cystic hepatic masses with the abovementioned US or CT appearance strongly raise the suspicion of liver abscess and potentially further complicate treatment of HCC. Serum AFP supports the suspicion of HCC although it is not always elevated, as in our case [6].

In our case, abdominal pain, septic-type fever, leucocytosis, and imaging study made us consider the diagnosis of hepatic abscess. In addition, medical history of ERCP strongly enforced this suspicion. Tumor markers are not specific. The fact that serum AFP was not raised did not favored the diagnosis of HCC. In contrast, Ca 19-9 may often result raised in pancreatic or cholangitic inflammations while Ca-125 is often elevated in cases of intraabdominal collections of fluid or ovarian cancer. In our case, ovarian primary involvement was excluded by US and CT scan. Blood cultures are not always positive in cases of liver abscesses. US-or CT-guided drainage are indicated for liver abscesses although multiple involvement made us consider the open drainage and biopsies. The diagnosis of HCC was enforced intraoperatively (shrinkage, darkbrown and firm hepatic ground) and confirmed by the histological examination.

## 4. Conclusion

Hepatic cystic mass with hyperpyrexia and leukocytosis without extrahepatic focus of inflammation must raise the suspicion of HCC. Ultrasound-guided biopsy for culture and tissue wall samples for histologic examination should be attempted in time in order to reach a diagnosis and plan the appropriate surgical or medical approach.
